# Rational Discourse on Virology and Pandemics

**DOI:** 10.1128/mbio.00313-23

**Published:** 2023-04-19

**Authors:** David Bahry

**Affiliations:** a Department of Biology, Carleton University, Ottawa, Ontario, Canada

**Keywords:** COVID-19, SARS-CoV-2, gain of function, laboratory leak, public trust in science, zoonosis

## Abstract

A group of 156 virologists, including American Society of Microbiology journal editors-in-chief, has recently published across three ASM journals a “call for rational discourse” on such important topics as the origin of SARS-CoV-2 and gain of function research (e.g., F. Goodrum et al., *mBio* 14:e0018823, 2023, https://doi.org/10.1128/mbio.00188-23). Here, I answer the call, arguing that the origin of SARS-CoV-2 is unknown; that continued premature downplaying of a possible laboratory origin, now accompanied by a denial that this was ever so dismissed, undermines public trust in science; and that the benefits from risky gain-of-function research-of-concern are fewer than Goodrum et al. imply.

## COMMENTARY

COVID-19 has now killed over six million people (1). We have not found the source of the virus responsible, SARS-CoV-2. Theories of natural spillover, or of a research-related accident, both remain plausible (2–4). Hypotheses include spillover from wildlife sold at the Huanan Seafood Market (HSM); a laboratory accident at the Wuhan Institute of Virology (WIV); spillover at another market; an accident at another laboratory; and direct infection of a human by a bat, whether a guano miner, a virologist collecting samples, or someone else.

Regardless of SARS-CoV-2’s origins, there are risks and benefits in virology. The creation and study of viruses with increased infectiousness or virulence is especially controversial (5–8).

Matters of public concern must be subject to transparent, inclusive deliberation and decision-making—both because a public facing risk must have a say in those risks and because these are crucial to public trust, which is crucial to promoting public health measures (9).

Goodrum et al. have recently published, across three ASM journals, a “call for rational discourse” on gain-of-function research and the origins of SARS-CoV-2 (10–12). Here, I argue that the origin of SARS-CoV-2 is unknown; that continued downplaying of a possible laboratory scenario, now accompanied by denial that this was ever so dismissed, undermines public trust in science; and that the benefits from risky gain-of-function research-of-concern are fewer than these authors imply.

## PUBLIC TRUST IN SCIENCE

Goodrum et al. agree that distrust in science is “disastrous.” Yet they fail to admit a role of the scientific establishment in contributing to broken trust—thereby undermining its repair.

Consider the well-known premature dismissal of the laboratory leak hypothesis (13). It is now within the pale to agree with Bloom et al. that “Theories of accidental release from a lab and zoonotic spillover both remain viable” (2) and the *Lancet* COVID-19 Commission that “The proximal origins of SARS-CoV-2 remain unknown” (3). This was not always so. In February 2020, a multiauthor letter to *The Lancet* (organized by a funder and close collaborator of WIV) disparaged laboratory leak as “conspiracy theories” (14, 15). In March 2020, a prominent letter to *Nature Medicine* by Andersen et al. (after a discreet teleconference to discuss their own early suspicions of engineering or serial passage with other virologists and the directors of the National Institutes of Health, the National Institute of Allergy and Infectious Disease, and the Wellcome Trust) said: “we do not believe that any type of laboratory-based scenario is plausible” (16, 17). Despite opposing voices from some scientists and journalists (18, 19), the narrative of laboratory leak as a fringe conspiracy theory dominated journalism, fact-checking services, and social media in 2020 and early 2021—including a period of outright censorship by Facebook of laboratory-modification hypotheses, “following consultations with leading health organizations” (13, 20).

This trend only changed after the publication in March 2021 of the joint World Health Organization (WHO)-China Study, which (pressured by China-approved members) had summarily dismissed laboratory leak as “extremely unlikely” (21, 22). At a briefing, WHO Director-General Tedros Adhanom Ghebreyesus disclaimed that he did “not believe that this assessment was extensive enough” (23). This was followed in May 2021 by Bloom et al.’s letter in *Science*, by many respected scientists; by articles in media such as *Bulletin of the Atomic Scientists*, *Current Affairs*, and the *New York Times*; and by Facebook ending its censorship of laboratory-modification hypotheses (2, 20, 24–26).

The overwhelming initial dismissal of a plausible hypothesis as a debunked conspiracy theory, and the surrounding lack of transparency, did great harm to public trust. However, far from acknowledging this, Goodrum et al. appear to *deny* it, alleging that “Most virologists have been open-minded about the possible origins of SARS-CoV-2.”

If scientists genuinely want the public’s trust, then we will acknowledge our errors and commit to do better—not deny what they and we all witnessed and remember.

## ORIGIN OF SARS-CoV-2

Though assenting that “each of these possibilities is plausible,” Goodrum et al. go on to claim that “currently the zoonosis hypothesis has the strongest supporting evidence.” This claim cites four references. One is misleading: Relman did not say that zoonosis had stronger support, he said that “none of these scenarios can be confidently ruled in or ruled out” (19). The rest are from a coauthor network centered on the first-draft authors of the *Nature Medicine* letter: Andersen, Rambaut, Holmes, and Garry (27–29). For limits of space, let us consider Worobey et al.

**FIG 1:**
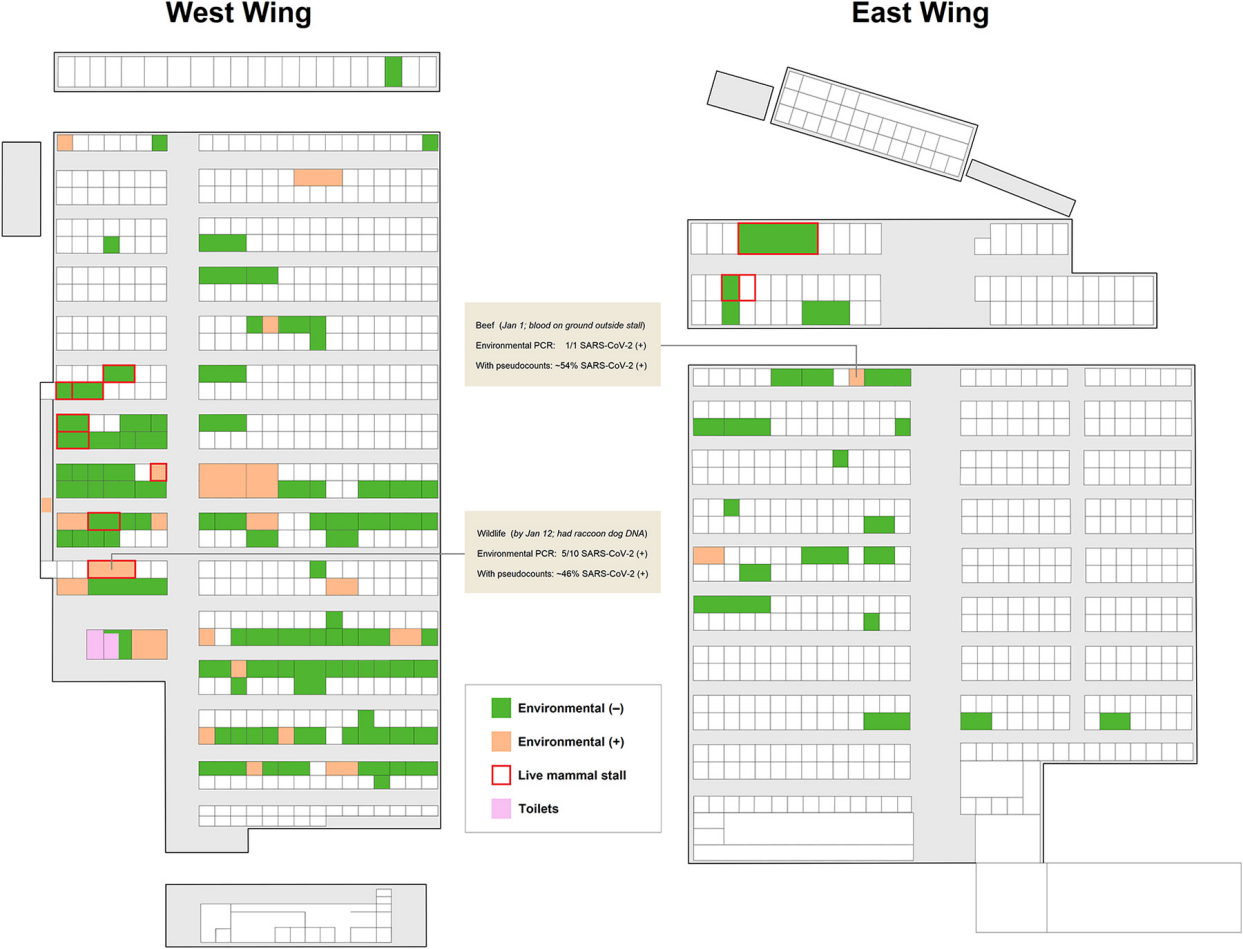
Ascertainment bias in the Huanan market. Stalls and samples adapted from Worobey et al. (28); sample color-coding from Gao et al. (32); toilets from Zhang et al. (43) based on photographic evidence (babarlelephant; http://babarlelephant.free-hoster.net/visiting-the-wuhan-seafood-market/); number of samples from Liu et al.’s Supplementary Table 1 (beef: east wing, street 9, vendor 22; wildlife: west wing, street 6, vendor 29-31-33) (66). Positivity estimated with pseudocounts as (*s*+0.077)/(*n*+1), taking into account uncertainty due to low *n*, and the 7.7% positivity base rate. There is ambiguity in Worobey et al.’s designation of some stalls as “unknown meat,” including a “+” stall that sold pig carcasses and preserved chopped pig legs (43). Most “+” and “−” stalls were in the southwestern corner, near where most wildlife was sold; wildlife stalls were also sampled far more heavily (66; cf. 30). Worobey et al. assumed all sampled stalls had been sampled equally, or at least with no heavier than 2× bias towards wildlife stalls (28, Fig. 4B–4D and Table S10). One of its senior authors, ignoring the clustering of “−” stalls, elsewhere said: “Most of the samples that later tested positive for SARS-CoV-2 were from the southwestern corner of the market” (60).

Worobey et al. (28) argue that the clustering of early cases around HSM, and within HSM the clustering of positive environmental samples in the southwestern corner of the market near most wildlife stalls, indicate that SARS-CoV-2 spilled over at HSM. Yet their arguments are circular, based on dismissing clear, known sampling bias in the data: authorities initially (understandably) assumed an HSM zoonosis, so that was what they largely *looked* for (30). This assumption guided both the early search for cases, near or potentially linked to HSM; and the environmental sampling within HSM, with special attention paid to the southwestern corner, near most wildlife stalls (Table 1; Fig. 1). Such a biased search would lead cases (especially ones with no market link) to appear to cluster near to and centered on HSM, and samples to appear to cluster in the southwestern corner, regardless of whether this reflected reality.

**TABLE 1:** Ascertainment bias in the search for cases and Huanan market environmental samples^*a*^

Quotation	Source
Recently, some medical institutions found that many pneumonia cases received were related to [Huanan] Seafood City. After receiving the report, the Municipal Health and Health Commission immediately carried out case searches and retrospective investigations **related to [Huanan] Seafood City** in medical and health institutions in the city. …	Wuhan Municipal Health Commission (61)
On December 29, 2019, a hospital in Wuhan admitted four individuals with pneumonia and recognized that all four had worked in the Huanan Seafood Wholesale Market, which sells live poultry, aquatic products, and several kinds of wild animals to the public. The hospital reported this occurrence to the local center for disease control (CDC), which lead [*sic*] Wuhan CDC staff to initiate a field investigation with a retrospective search for pneumonia patients **potentially linked to the market**. … A probable case of [Viral Pneumonia of Unknown Etiology] was defined as a surveillance VPUE case or an illness of unknown etiology fulfilling the first three surveillance VPUE case criteria **with a history of exposure to the Huanan Seafood Wholesale Market** in Wuhan or any other VPUE case. …	China CDC, Hubei CDC, Wuhan CDC (62)
30 Dec: A city-wide case screening was conducted targeting people with pneumonia of unknown origin, abnormal blood routine test (normal WBC, lymphocytopenia), and **exposure history with Huanan market**. … 31 Dec: Continued epidemiology surveillance **at several hospitals (close to Huanan market), Huanan market and the neighbourhood of Huanan market**. …	Joint WHO-China Study (63)
The range of in-market sampling covered: (1) environmental samples from stalls related to early cases; … (3) environmental samples in the east wing of the market were collected according to blocks; … (5) **environmental samples from stalls that sold livestock, poultry, farmed wildlife** … (10) public toilets, public activity rooms and other places where people gathered in the market. …	Joint WHO-China Study (21)

a The Joint WHO-China Study also reported on a retrospective analysis of influenza-like illnesses which in principle could have reduced the bias (p. 47–49); however, they did not find any cases, and disclaimed that this could have been due to the stringent requirement of severe symptoms and to the long delay between illness and serological testing (21). Worobey et al. said: “the geographical association of early COVID-19 cases with the Huanan market is unlikely to have been the result of ascertainment bias” (28). One of its senior authors elsewhere said: “there’s no evidence of widespread sampling bias” (60).

Although Worobey et al. purport to test for “robustness” of their results to sampling bias, their tests fail (30). For instance, they in effect address false positives near HSM, by dropping cases nearest to HSM from the data. But the issue was false negatives: cases missed due to *not* being near HSM. This is as fallacious as surveying New Yorkers; dropping the 68% most central ones from the data; and concluding from the remaining 32% of New Yorkers that most of humanity lives near to and centered on Central Park.

Although the first-discovered few cases, before the biased search began, included market-linked cases (31), this does not prove a HSM spillover. Gao et al., who searched HSM for environmental samples, concluded in a preprint “that SARS-CoV-2 might have derived from Homo sapiens in the HSM,” which acted as a superspreader “due to the high number of visitors every day” (32). A number of peer-reviewed studies give evidence of SARS-CoV-2 prior to the market cases (33, 34), even as early as October or September (35–40). While Worobey et al. (and their companion paper by Pekar et al. [41]) may dispute these findings, no scientific consensus can yet be presumed.

Worobey et al. and Pekar et al. were also promptly criticized by scientists in a number of preprints and eLetters (30, 42–47), one of them also recently published in a journal (48). To dismiss these due to not being published as Technical Comments in *Science* would be a confusion. *Science*’s information-for-authors page advises: “In the case of rapidly developing research fields (such as COVID-19), Technical Comment submissions may be directed to eLetters to allow a faster exchange of ideas” (49).

Although HSM spillover is one plausible scenario, the case is very far from dispositive.

### Addendum 1.

As I revise this Comment, a preprint has been posted, again from the same coauthor network, after being heralded by *The Atlantic* as “the strongest evidence yet” of an HSM origin; statements from the WHO were more sober (50–53). Crits-Christoph et al. analyzed a portion of Gao et al.’s briefly uploaded HSM metagenomic data (50). These researchers found that animal-product stalls had animal genetic material, including abundant cattle DNA in a beef stall, fish DNA on a fish packaging surface, and raccoon dog DNA in the presumptive raccoon dog cart. I agree with Dr. Tedros that, while all data are valuable, this does not settle the question of how the pandemic began (52).

### Addendum 2.

As this Comment goes to press, two further developments have occurred. First, *Science* has now discontinued Technical Comments entirely (64). Second, a revision of the Gao et al. preprint was posted by Liu et al., followed by publication in *Nature*, along with more complete data. As before, they emphasize that HSM may only have been an amplifier; they also explicitly highlighted sampling bias (65, 66). As well as more wildlife stalls being sampled, these stalls were also sampled far more heavily (66, Supplementary Table 1; cf. 30). For instance, blood outside a beef stall was sampled once, while the raccoon dog-linked stall was sampled ten times in early January alone (Fig. 1); ultimately two hundred-and-twenty samples were related to the latter, including from surfaces, water drains, and animals including hares and hedgehogs, including at the supplying warehouse.

## GAIN OF FUNCTION

“Gain of function research” (GOF) is a broad term, referring to the acquisition of any trait by any organism. It is sometimes used as a shorthand for risky “gain-of-function research-of-concern” (GOFROC), expected to enhance a pathogen’s transmissibility, virulence, or host range with risk to humans (7, 8). We must also distinguish whether research arguably or clearly *is* GOFROC, versus whether it is currently *recognized* as such and effectively regulated under any particular regulatory framework, such as the current U.S. regulatory framework for enhanced potential pandemic pathogens (enhanced PPP), now under review for likely revision (54).

Goodrum et al. defend broad GOF, emphasizing that it has resulted in U.S. Food and Drug Administration (FDA)-approved products, such as (replication-incompetent) adenovirus-vector COVID-19 vaccines expressing SARS-CoV-2 spike. This is a red herring; there was never any concern of a Johnson & Johnson pandemic. In their Table 2 (11), no experiment creating or enhancing a PPP led to any FDA-approved product.

They defend the creation of mammal-transmissible H5N1 bird flu (55, 56) and the insertion of a bat coronavirus spike into SARS (57) as establishing that H5N1 and bat coronaviruses threaten humans. Yet this research did not prevent COVID-19, nor did it lead to any FDA-approved product.

In their abstract, they disparage concerns that well-intentioned, risky research may even have *caused* the pandemic (58), claiming that a “small but vocal group” has conflated “legitimate questions about safely conducting virus-related research with uncertainties over the origins of SARS-CoV-2.” This seems misleading: their main text admits that laboratory leak is plausible.

The primary genuine benefit of GOFROC is not any current application, but as basic research. GOFROC is generally informative about viruses, and knowledge can in general yield unpredictable benefits: *T. aquaticus* was not discovered in order to invent PCR, nor nuclear magnetic resonance in order to invent the MRI machine. Researchers whose conviction is that this justifies GOFROC should be explicit about their reasons, when communicating with the public. Only then can we deliberate together how to weigh the public benefits of GOFROC relative to alternative approaches, against the risks of lab accidents (7, 9, 59).

### Conclusion.

Goodrum et al. plea for scientific experts, not legislation, to decide on biosafety policy. But for self-governing virology or public health science to be effective, it must have the public’s trust.

Unfortunately, by continuing to prematurely downplay a hypothesis even as they deny that it was ever so dismissed, and obscuring debates over risky GOFROC by appealing to the benefits of safe research of no concern, Goodrum et al. inspire little confidence that they take seriously the risks involved in GOFROC, nor that they take seriously the necessity of transparency and inclusion, to repair public trust and promote public health.
